# Biphenyl-4,4′-dicarb­oxy­lic acid *N*,*N*-dimethyl­formamide monosolvate

**DOI:** 10.1107/S1600536810030515

**Published:** 2010-08-04

**Authors:** Søren Jakobsen, David Stephen Wragg, Karl Petter Lillerud

**Affiliations:** a inGAP Centre for Research Based Innovation, Department of Chemistry, University of Oslo, 0315 Oslo, Norway

## Abstract

Biphenyl-4,4′-dicarb­oxy­lic acid was recrystallized from *N*,*N*-dimethyl­formamide (DMF) yielding the title compound, C_14_H_10_O_4_·2C_3_H_7_NO. The acid mol­ecules are located on crystallographic centres of inversion and are hydrogen bonded to DMF mol­ecules. These hydrogen-bonded units form infinite chains although there is no inter­action between the methyl groups of neighboring DMF mol­ecules.

## Related literature

The title compound is a popular linker for the synthesis of metal-organic framework materials, for example IRMOF 10 (Eddaoudi *et al.*, 2002 ▶) and UIO-67 (Cavka *et al.*, 2008 ▶).
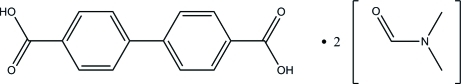


## Experimental

### 

#### Crystal data

C_14_H_10_O_4_·2C_3_H_7_NO
*M* *_r_* = 388.41
Triclinic, 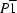

*a* = 7.666 (7) Å
*b* = 7.774 (7) Å
*c* = 9.099 (8) Å
α = 88.549 (10)°
β = 73.596 (10)°
γ = 65.208 (7)°
*V* = 469.6 (7) Å^3^
*Z* = 1
Mo *K*α radiation
μ = 0.10 mm^−1^
*T* = 150 K
0.2 × 0.2 × 0.1 mm

#### Data collection

Bruker APEX CCD area-detector diffractometer
Absorption correction: multi-scan (*SADABS*; Sheldrick, 2004 ▶) *T*_min_ = 0.980, *T*_max_ = 0.990
3968 measured reflections
2136 independent reflections
1635 reflections with *I* > 2σ(*I*)
*R*_int_ = 0.015

#### Refinement

*R*[*F* ^2^ > 2σ(*F* ^2^)] = 0.044
*wR*(*F* ^2^) = 0.175
*S* = 1.11
2136 reflections
129 parameters
H-atom parameters constrained
Δρ_max_ = 0.40 e Å^−3^
Δρ_min_ = −0.32 e Å^−3^

### 

Data collection: *SMART* (Bruker, 2001 ▶); cell refinement: *SAINT* (Bruker, 2001 ▶); data reduction: *SAINT*; program(s) used to solve structure: *SHELXS97* (Sheldrick, 2008 ▶); program(s) used to refine structure: *SHELXL97* (Sheldrick, 2008 ▶); molecular graphics: *DIAMOND* (Brandenburg, 2006 ▶); software used to prepare material for publication: *SHELXL97* and *enCIFer* (Allen *et al.*, 2004 ▶).

## Supplementary Material

Crystal structure: contains datablocks I, global. DOI: 10.1107/S1600536810030515/bt5277sup1.cif

Structure factors: contains datablocks I. DOI: 10.1107/S1600536810030515/bt5277Isup2.hkl

Additional supplementary materials:  crystallographic information; 3D view; checkCIF report

## Figures and Tables

**Table 1:** Hydrogen-bond geometry (Å, °)

*D*—H⋯*A*	*D*—H	H⋯*A*	*D*⋯*A*	*D*—H⋯*A*
O2—H2*A*⋯O3^i^	0.82	1.76	2.575 (2)	172

Symmetry code: (i) 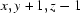.

**Fig. 1.**
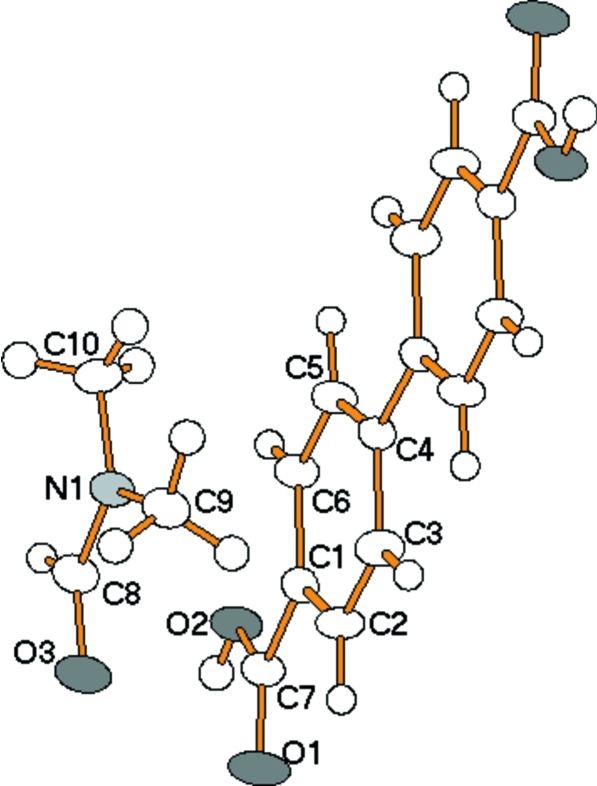
The molecular structure of (I), with atom labels and 50% probability displacement ellipsoids for non-H atoms. Unlabeled atoms are related to the labeled ones by an inversion centre.

**Fig. 2.**
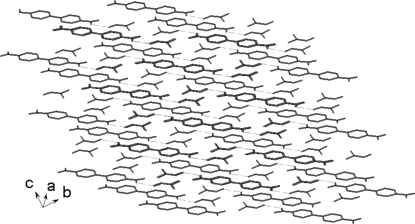
The packing of (I), showing the hydrogen bonded chains. Hydrogen atoms are omitted and hydrogen bonds are shown as dashed lines.

**Fig. 3.**
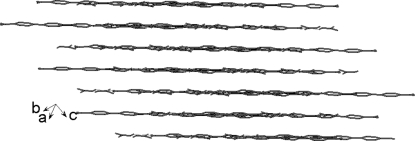
The packing of (I), showing the layers formed by the chains. Hydrogen atoms are omitted and hydrogen bonds are shown as dashed lines.

## supplementary crystallographic information

### Comment

The title compound, (I) (Fig. 1), which is a popular linker for the synthesis of metal-organic framework materials, for example IRMOF 10 (Eddaoudi *et al.*, 2002) and UIO-67 (Cavka *et al.*, 2008), comprises units of one biphenyl-4,4'-dicarboxylic acid molecule hydrogen bonded to two DMF molecules *via* O—H···O links. These units pack as chains (Fig. 2), although there is no interaction between the methyl groups of neighboring DMF molecules. The chains are arranged in layers with no stacking interactions between the benzene rings (Fig. 3).

### Experimental

Biphenyl-4,4'-dicarboxylic acid and *N*,*N*-Dimethylformamide (DMF) were purchased from Sigma-Aldrich and used without further purification. 1.0 g Biphenyl-4,4'dicarboxylic acid was suspended in 100 ml DMF and heated to 100°C. DMF was added in small portions until the acid had just dissolved (app. 50 ml) and the solution left in aluminium foil over night for slow cool-down to RT. Filtration of the now 125 ml DMF suspension yielded 0.57 g white powder of Biphenyl-4,4-dicarboxylic acid after drying under vacuum. The mother liquor was placed at 5°C over night which gave a small amount of colourless crystals, which gave the structure presented here.

### Refinement

Hydrogen atoms were placed in ideal positions and refined with a riding model with C-H = 0.93Å and U(H)=1.2U_eq_(C) or with C-H = 0.96Å and U(H)=1.5U_eq_(C_methyl_).

### Crystal data

**Table:**

C_14_H_10_O_4_·2C_3_H_7_NO	*Z* = 1
*M**_r_* = 388.41	*F*(000) = 206
Triclinic, *P*1	*D*_x_ = 1.374 Mg m^−^^3^
*a* = 7.666 (7) Å	Mo *K*α radiation, λ = 0.71073 Å
*b* = 7.774 (7) Å	Cell parameters from 1412 reflections
*c* = 9.099 (8) Å	θ = 2.4–28.2°
α = 88.549 (10)°	µ = 0.10 mm^−^^1^
β = 73.596 (10)°	*T* = 150 K
γ = 65.208 (7)°	Prism, colourless
*V* = 469.6 (7) Å^3^	0.2 × 0.2 × 0.1 mm

### Data collection

**Table:**

Bruker APEX CCD area-detector diffractometer	2136 independent reflections
Radiation source: sealed tube	1635 reflections with *I* > 2σ(*I*)
graphite	*R*_int_ = 0.015
phi and ω scans	θ_max_ = 28.8°, θ_min_ = 2.9°
Absorption correction: multi-scan (*SADABS*; Sheldrick, 2004)	*h* = −9→10
*T*_min_ = 0.980, *T*_max_ = 0.990	*k* = −10→10
3968 measured reflections	*l* = −12→12

### Refinement

**Table:**

Refinement on *F*^2^	Primary atom site location: structure-invariant direct methods
Least-squares matrix: full	Secondary atom site location: difference Fourier map
*R*[*F*^2^ > 2σ(*F*^2^)] = 0.044	Hydrogen site location: inferred from neighbouring sites
*wR*(*F*^2^) = 0.175	H-atom parameters constrained
*S* = 1.11	*w* = 1/[σ^2^(*F*_o_^2^) + (0.1177*P*)^2^] where *P* = (*F*_o_^2^ + 2*F*_c_^2^)/3
2136 reflections	(Δ/σ)_max_ < 0.001
129 parameters	Δρ_max_ = 0.40 e Å^−^^3^
0 restraints	Δρ_min_ = −0.32 e Å^−^^3^

### Special details

**Table:**

Geometry. All s.u.'s (except the s.u. in the dihedral angle between two l.s. planes) are estimated using the full covariance matrix. The cell s.u.'s are taken into account individually in the estimation of s.u.'s in distances, angles and torsion angles; correlations between s.u.'s in cell parameters are only used when they are defined by crystal symmetry. An approximate (isotropic) treatment of cell s.u.'s is used for estimating s.u.'s involving l.s. planes.
Refinement. Refinement of *F*^2^ against ALL reflections. The weighted *R*-factor *wR* and goodness of fit *S* are based on *F*^2^, conventional *R*-factors *R* are based on *F*, with *F* set to zero for negative *F*^2^. The threshold expression of *F*^2^ > 2σ(*F*^2^) is used only for calculating *R*-factors(gt) *etc*. and is not relevant to the choice of reflections for refinement. *R*-factors based on *F*^2^ are statistically about twice as large as those based on *F*, and *R*- factors based on ALL data will be even larger.

### Fractional atomic coordinates and isotropic or equivalent isotropic displacement parameters (Å^2^)

**Table:**

	*x*	*y*	*z*	*U*_iso_*/*U*_eq_	
C1	0.1394 (2)	0.7816 (2)	0.21617 (19)	0.0217 (4)	
C2	−0.0126 (3)	0.8677 (2)	0.3535 (2)	0.0260 (4)	
H2	−0.0795	0.9999	0.3711	0.031*	
C3	−0.0653 (3)	0.7578 (2)	0.4645 (2)	0.0265 (4)	
H3	−0.1653	0.8178	0.5567	0.032*	
C4	0.0289 (2)	0.5590 (2)	0.44054 (18)	0.0208 (4)	
C5	0.1794 (3)	0.4743 (2)	0.30105 (19)	0.0252 (4)	
H5	0.2442	0.3421	0.2816	0.030*	
C6	0.2336 (2)	0.5847 (2)	0.1908 (2)	0.0251 (4)	
H6	0.3344	0.5255	0.0989	0.030*	
C7	0.1957 (2)	0.9059 (2)	0.10229 (19)	0.0240 (4)	
O1	0.1055 (2)	1.07728 (18)	0.11935 (16)	0.0363 (4)	
O2	0.35493 (18)	0.80786 (17)	−0.01629 (14)	0.0282 (3)	
H2A	0.3804	0.8814	−0.0753	0.042*	
O3	0.44518 (19)	0.01439 (18)	0.77730 (15)	0.0333 (4)	
N1	0.3688 (2)	0.3198 (2)	0.72336 (17)	0.0249 (4)	
C8	0.3490 (3)	0.1893 (2)	0.8138 (2)	0.0267 (4)	
H8	0.2566	0.2312	0.9116	0.032*	
C9	0.5089 (3)	0.2649 (3)	0.5689 (2)	0.0292 (4)	
H9A	0.4432	0.3379	0.4976	0.044*	
H9B	0.5531	0.1317	0.5410	0.044*	
H9C	0.6229	0.2888	0.5657	0.044*	
C10	0.2551 (3)	0.5215 (2)	0.7753 (2)	0.0304 (4)	
H10A	0.1787	0.5833	0.7065	0.046*	
H10B	0.3461	0.5763	0.7765	0.046*	
H10C	0.1653	0.5386	0.8773	0.046*	

### Atomic displacement parameters (Å^2^)

**Table:**

	*U*^11^	*U*^22^	*U*^33^	*U*^12^	*U*^13^	*U*^23^
C1	0.0211 (8)	0.0194 (8)	0.0226 (8)	−0.0075 (6)	−0.0056 (6)	0.0033 (6)
C2	0.0264 (8)	0.0157 (8)	0.0281 (9)	−0.0054 (6)	−0.0020 (7)	0.0009 (6)
C3	0.0262 (8)	0.0204 (8)	0.0242 (8)	−0.0076 (6)	0.0015 (6)	−0.0006 (6)
C4	0.0199 (8)	0.0187 (8)	0.0218 (8)	−0.0066 (6)	−0.0061 (6)	0.0033 (6)
C5	0.0274 (8)	0.0155 (8)	0.0243 (8)	−0.0045 (6)	−0.0029 (7)	0.0016 (6)
C6	0.0267 (8)	0.0178 (8)	0.0224 (8)	−0.0054 (6)	−0.0012 (6)	0.0010 (6)
C7	0.0244 (8)	0.0186 (8)	0.0247 (8)	−0.0069 (6)	−0.0047 (6)	0.0029 (6)
O1	0.0389 (8)	0.0173 (7)	0.0360 (8)	−0.0057 (6)	0.0031 (6)	0.0042 (5)
O2	0.0307 (7)	0.0185 (6)	0.0252 (7)	−0.0071 (5)	0.0006 (5)	0.0045 (5)
O3	0.0358 (7)	0.0216 (7)	0.0326 (7)	−0.0083 (5)	−0.0024 (6)	0.0065 (5)
N1	0.0260 (7)	0.0190 (7)	0.0265 (7)	−0.0084 (6)	−0.0053 (6)	0.0036 (6)
C8	0.0254 (8)	0.0238 (9)	0.0260 (9)	−0.0080 (7)	−0.0047 (7)	0.0051 (6)
C9	0.0308 (9)	0.0251 (9)	0.0274 (9)	−0.0108 (7)	−0.0045 (7)	0.0061 (7)
C10	0.0342 (9)	0.0199 (9)	0.0342 (9)	−0.0095 (7)	−0.0090 (7)	−0.0001 (7)

### Geometric parameters (Å, °)

**Table:**

C1—C6	1.383 (3)	C7—O2	1.324 (2)
C1—C2	1.390 (2)	O2—H2A	0.8200
C1—C7	1.497 (2)	O3—C8	1.244 (2)
C2—C3	1.389 (3)	N1—C8	1.321 (2)
C2—H2	0.9300	N1—C10	1.449 (2)
C3—C4	1.396 (3)	N1—C9	1.451 (2)
C3—H3	0.9300	C8—H8	0.9300
C4—C5	1.398 (2)	C9—H9A	0.9600
C4—C4^i^	1.493 (3)	C9—H9B	0.9600
C5—C6	1.392 (2)	C9—H9C	0.9600
C5—H5	0.9300	C10—H10A	0.9600
C6—H6	0.9300	C10—H10B	0.9600
C7—O1	1.206 (2)	C10—H10C	0.9600
			
C6—C1—C2	118.77 (15)	O2—C7—C1	112.84 (15)
C6—C1—C7	122.67 (15)	C7—O2—H2A	109.5
C2—C1—C7	118.56 (16)	C8—N1—C10	121.51 (15)
C3—C2—C1	120.52 (16)	C8—N1—C9	120.74 (15)
C3—C2—H2	119.7	C10—N1—C9	117.75 (14)
C1—C2—H2	119.7	O3—C8—N1	124.59 (17)
C2—C3—C4	121.27 (16)	O3—C8—H8	117.7
C2—C3—H3	119.4	N1—C8—H8	117.7
C4—C3—H3	119.4	N1—C9—H9A	109.5
C3—C4—C5	117.62 (14)	N1—C9—H9B	109.5
C3—C4—C4^i^	121.26 (18)	H9A—C9—H9B	109.5
C5—C4—C4^i^	121.12 (18)	N1—C9—H9C	109.5
C6—C5—C4	120.95 (15)	H9A—C9—H9C	109.5
C6—C5—H5	119.5	H9B—C9—H9C	109.5
C4—C5—H5	119.5	N1—C10—H10A	109.5
C1—C6—C5	120.84 (16)	N1—C10—H10B	109.5
C1—C6—H6	119.6	H10A—C10—H10B	109.5
C5—C6—H6	119.6	N1—C10—H10C	109.5
O1—C7—O2	124.29 (16)	H10A—C10—H10C	109.5
O1—C7—C1	122.87 (16)	H10B—C10—H10C	109.5
			
C6—C1—C2—C3	1.5 (3)	C7—C1—C6—C5	178.71 (15)
C7—C1—C2—C3	−177.92 (15)	C4—C5—C6—C1	−0.2 (3)
C1—C2—C3—C4	−1.4 (3)	C6—C1—C7—O1	175.06 (16)
C2—C3—C4—C5	0.4 (3)	C2—C1—C7—O1	−5.5 (3)
C2—C3—C4—C4^i^	−179.51 (17)	C6—C1—C7—O2	−5.8 (2)
C3—C4—C5—C6	0.4 (3)	C2—C1—C7—O2	173.65 (15)
C4^i^—C4—C5—C6	−179.67 (17)	C10—N1—C8—O3	−178.38 (16)
C2—C1—C6—C5	−0.7 (3)	C9—N1—C8—O3	0.5 (3)

Symmetry codes: (i) −*x*, −*y*+1, −*z*+1.

### Hydrogen-bond geometry (Å, °)

**Table:**

*D*—H···*A*	*D*—H	H···*A*	*D*···*A*	*D*—H···*A*
O2—H2A···O3^ii^	0.82	1.76	2.575 (2)	172.

Symmetry codes: (ii) *x*, *y*+1, *z*−1.
